# The Effect of Radionuclide and Chemical Contamination on Morphological and Anatomical Parameters of Plants

**DOI:** 10.3390/plants13202860

**Published:** 2024-10-12

**Authors:** Alyona Yankauskas, Natalya Larionova, Anton Shatrov, Anna Toporova

**Affiliations:** Institute of Radiation Safety and Ecology, National Nuclear Center of the Republic of Kazakhstan, Kurchatov City 180010, Kazakhstan; alyona.yankauskas@mail.ru (A.Y.); anton-shatrov@mail.ru (A.S.); toporova@nnc.kz (A.T.)

**Keywords:** radionuclides, elements, plants, morpho-anatomical parameters, dose rate

## Abstract

This article presents the results of a study of the influence of radionuclide and chemical pollution on the morphological and anatomical parameters of *Calamagróstis epigéjos* plants growing in the territory of “Degelen” at the Semipalatinsk Test Site (STS). Quantitative data of morphological and anatomical parameters are given, and the content of radionuclides and toxic elements in samples of plants obtained. Statistical processing of the obtained data was conducted. The results revealed that elevated concentrations of radionuclides ^137^Cs and ^90^Sr, and the calculated absorbed dose, do not have effects on plants. Changes in the anatomical parameters of leaves and stems were observed at elevated concentrations of the elements: for leaves—Al, Pb, Sr, U, Ni, Rb, Sm; for stems—Al, Cr, Cd, U, Cu, Be, Ni, Sm, Fe. The mesophyll of the leaves and the epidermis of the stems were the most exposed to toxic elements. The data of the anatomical parameters are recommended to be used as indicative parameters of plants grown in chemically contaminated areas.

## 1. Introduction

Nuclear tests have resulted in the formation of high radiation doses to biota. The effect of ionizing radiation on plants and animals is evident at all levels of biological organization—from the molecular and cell levels to the levels of population and ecosystem. Variations can be registered in plants and animals at a relatively low dose at the cellular level, whereas no visible change at the levels of populations and ecosystems is marked [1]. Until recently, the study of the effects of ionizing radiation on wildlife was limited mainly to the accumulation of quantitative data on the level of the dose–effect relationship for different organisms. Currently, the solution to this problem is linked to the establishment of regularities of radiation effects on the biosphere or ecological communities. The cell-tissue level (morphological and anatomical) is the second most radiosensitive after the cytogenetic and molecular levels. Most frequently, these are used as the test criteria for the growth of annual shoots and the development of assimilating organs (needles and leaves) [2].

Variations in morphological and anatomical structure were observed in plants growing in conditions of radiation exposure [3,4,5]. Chronic exposure to toxicants causes serious changes in the anatomical structure of plant leaves [6,7]. The effect of chronic ionizing radiation at low doses on morphological and anatomical structure has been insufficiently studied [8]. In turn, the impact of radiation on flora and fauna depends on the dose and the radiosensitivity of the organisms, as well as on the influence of some other factors [9]. Specific morpho-anatomical and physiological characteristics of plants may occur as a result of adaptation characteristics in adverse growing conditions [10,11]. Stressful conditions can have a significant effect on the differentiation of sclerenchyma fibers in the leaves of cereal plants [12].

According to research conducted previously, as *Stipa capillata* responds by increasing the exposure dose with the emergence of adaptive characteristics, i.e., the increase in the thickness of epidermis, sclerenchyma layer, and the number of conductive bundles, leaves increase the thickness of the cells’ upper and lower epidermis, the thickness of mesophyll, and the area of conducting bundles [13]. In the anatomical structure of the root of *Potentilla bifurca*, the thickness of periderm, the medullary rays, and the area of the ksylem vessels observed in the inhibition of growth increased [14].

The negative impact on the natural environment increases if it simultaneously presents several polluting substances. One of the urgent environmental problems for Kazakhstan is the pollution of the territory with heavy metals. The extent of pollution and the impact on biological objects of environmental contaminants have a special place and are among the most common and dangerous environmental pollutants for biota, as characterized by high toxicity, and mutagenic and carcinogenic effects [15]. Some heavy metals such as Cu, Zn, Mn, and Fe are required by plants as structural and catalytic components of proteins and enzymes, and they are usually called microelements. Unclaimed heavy metals such as Pb, Cd, Hg, and Cr do not play an important role in metabolic processes and are considered toxic [16]. High concentrations of heavy metals in the soil have a negative impact on the structure of plants [17,18,19,20]. It was noted that in areas contaminated with heavy metals, leaf thickness is less than in “background” territories [21,22,23]. In consequence of the reduction in anatomical and morphological parameters of plants, hidden damage and physiological disorders are marked [12,24]. A decrease has been recorded in the thickness of mesophyll and the parenchyma of the leaves of tansy (*Tanacetum vulgare* L.) growing in conditions of environmental pollution with heavy metals [25].

Different species and varieties of plant differ in their ability to accumulate heavy metals at the same concentrations of elements in the environment [26]. In studying the effect of lead and cadmium on plants, a lockup of plant growth was observed, the area of the leaf blade was reduced by 20–40%, and the deformation of leaves of the cultivated plant species and a significant change of chlorophyll and intensity of photosynthesis were observed [27]. Nickel inhibits the germination of seeds and the growth of many crops [28,29]. The combination of Ni and NaCl in seedlings of cabbage seeds showed a significant decline in growth, leaf water potential, photosynthetic pigments and activity, lipid peroxidation, and the activity of anti-oxidative enzymes [30]. The content of heavy metals in the aerial part increases sharply when soil is contaminated, and this leads to the inhibition of growth and the development and basic physiological functions of plants [26].

The Republic of Kazakhstan has one of the three world’s largest test sites—the Semipalatinsk Test Site (STS). The total area of the STS is 18,500 km^2^. Nuclear tests were conducted at test locations. The ecological situation in the territory of the STS is marked by the combination of radiation [31,32,33] and “non-radiation” factors [34,35]. The specified area is named as “Degelen” [36,37,38]. For many years, areas adjacent to the mountain range “Degelen” were studied [39], including more than 40 chemical elements, and an abnormally high content of some elements was identified. The pattern of the areal distribution of element concentrations is differentiated and expressed at irregularly shaped local spots in all areas.

In the territory of the Semipalatinsk Test Site, there are minerals such as W, Be, Mo, Zr, Sb, and Sn. A group of greisen bodies was revealed to form a tungsten ore field located on the Western slope of the mountains of Degelen. Ores contain wolframite, hematite, pyrite, sphalerite, scheelite, molybdenite, beryl, and other minerals. Thus, heavy and toxic elements are expected to accumulate in abnormal quantities in the soil, water, and plants. From this point of view, the most unique is tunnel No. 504 in the “Degelen” area. The platform gallery is characterized by a high content of elements and radionuclides. The contents of rare earth elements in the water stream outflowing from this gallery ranges from 8.0 ± 0.8 µg/L to 890 ± 85 µg/L, and the concentrations of Al, Mn, and Zn are comparable with those of macrocomponents [35]. Levels of ^137^Cs and ^90^Sr in plants range within *n* × 10^3^ *n* × 10^4^ Bq/kg [36].

Previously, research was conducted into the morpho-anatomical structure of plants (*Phragmites australis*) growing only in conditions of chronic exposure to ionizing radiation in the territory of the STS [40]. The aim of this work was to study the morpho-anatomical parameters of plants (*Calamagróstis epigéjos*) growing in conditions of combined radionuclide and chemical contaminants for the first time in the territory of the STS.

## 2. Results and Discussion

### 2.1. Morphological Parameters of Plants

The original data of morphological measurements are presented in Table 1.

### 2.2. Anatomical Parameters of Plants

Average values of anatomical parameters of plants are presented in Table 2. For each of the study sites we conducted about 160 measurements. For each site, measurements ranged from 4 to 32 µSv/h, and the flux density of β-particles ranged from 10 to 1000 particles/min × cm^2^.

The total produced about 600 measurements of four anatomical parameters of stems and leaves at 10 research sites.

**Figure 1:**
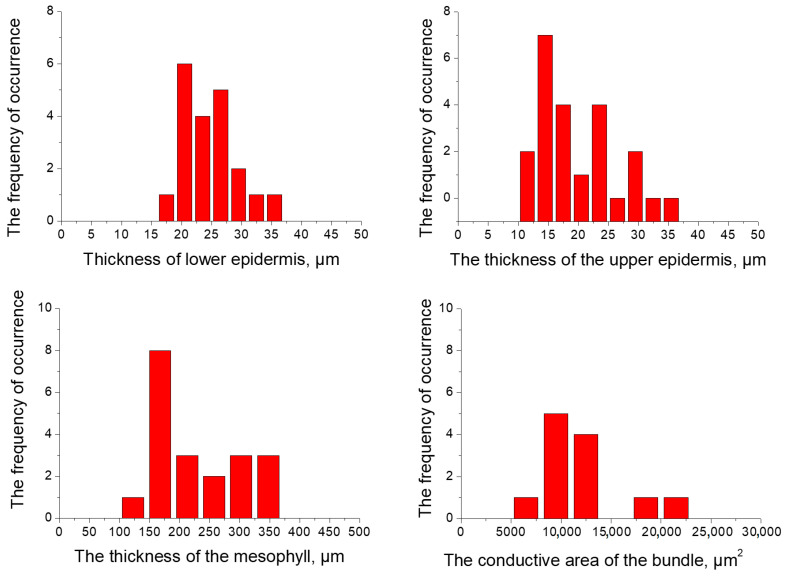
The variation distribution series values of the anatomical parameters of the leaves of *Calamagróstis epigéjos* for a single typical research site.

Statistical data processing was typical for all sites, as was the distribution of the values of the anatomical parameters of the sheet obtained. The distribution of values shown in the example of a single site graphically (Figure 1).

The distribution of values of the anatomical parameters are asymmetric. The distribution of values of the thickness of the epidermis and the area of the conductive bundle are asymmetrical (a measure of asymmetry greater than zero).

Table 3 presents ranges of anatomical parameters of the leaf of the plant, the arithmetic means calculated, and the median, standard deviation, coefficient of variation, and index of asymmetry.

The table shows that the values of the anatomical parameters of the leaf are approximately at the same level. The value of the median in most cases is almost identical with the average value, indicating no outliers. The coefficients of variation are small, despite the relatively wide ranges of values of the anatomical parameters of the leaf, i.e., the bulk of the values laid the “heap”. The maximum variation is observed for the anatomical parameters of the first research facilities and does not exceed 31%.

**Figure 2:**
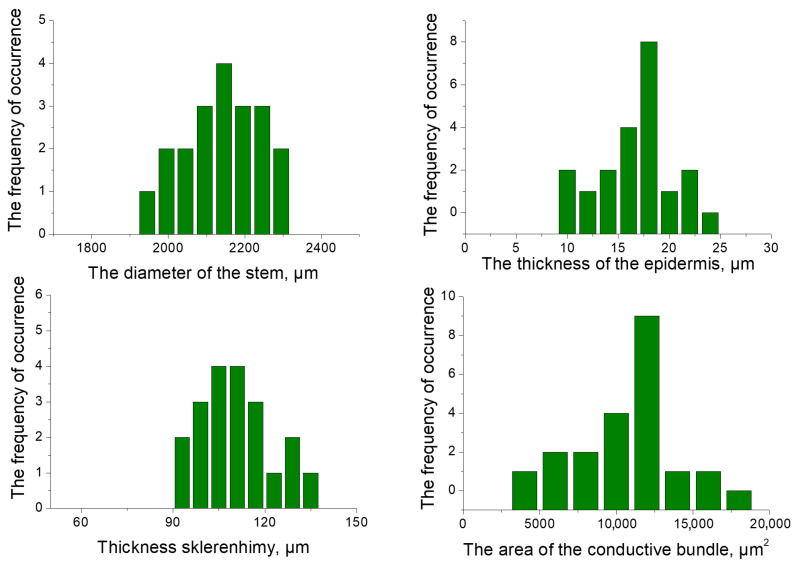
The variation distribution series values of the anatomical parameters of the stems of *Calamagróstis epigéjos* for a single typical research site.

Typical for all sites are the distribution of values of the anatomical parameters of stem, as presented (Figure 2).

Table 4 shows the ranges of anatomical parameters of stem plants, the calculated arithmetic means, and the median, standard deviation, and coefficient of variation and index of asymmetry.

### 2.3. Radionuclide Concentration in Plants

Table 5 presents the results of the radionuclide composition of plants: the natural radionuclides ^40^K, ^226^Ra, and ^232^Th, the transuranic radionuclides ^241^Am and ^239+240^Pu, and the fission products ^137^Cs and ^90^Sr. All results are presented on the fresh weight of the sample plants.

The content of natural and transuranic radionuclides in most cases is below the detection limit. The contents of the radionuclide ^40^K is from 170 ± 30 to 300 ± 60 Bq/kg. High values of the specific activity established for the radionuclides ^137^Cs and ^90^Sr. The specific activity of ^137^Cs in the investigated plants varies from 1.1 × 10^3^ to 4.9 × 10^4^ Bq/kg, ^90^Sr from 2.2 × 10^3^ to 6.2 × 10^4^ Bq/kg, and ^241^Am and ^239+240^Pu in the vast majority are below the detection limit of the equipment used.

### 2.4. Evaluation of Internal Exposure Dose of Plants

The calculation of the dose of internal exposure of plants from each radionuclide was conducted on the basis of the laboratory analyses of samples of vegetation and dose coefficients. The values of the dose rates of the internal irradiation of plants are presented in Table 6.

According to the table, the values of absorbed dose rate at the sites range from 40 to 760 µGy/day.

### 2.5. Content of Elements in Plants

Concentrations of toxic elements in the samples of plants are presented in Table 6. The method of comparison of the obtained results occurring with typical concentrations of elements in plants according to the literature is used to identify the elements–pollutants [42,43,44,45,46].

The results revealed that, of the 21 studied elements, those exceeding common concentrations in plants observed were 15 (Table 7).

The content of lanthanides in the samples exceeds the normal naturally occurring concentrations by 30–50 times or more, U is approximately 25–260, Li and Cr are 10–20, and Mn, Cd, and Al are 2–10 times more [46].

### 2.6. The Identification of Dependencies between the Investigated Parameters

Table 8 shows correlation coefficients calculated to detect the possible impact of radionuclides on anatomical parameters of plants.

The results of the calculation of correlation coefficients between the investigated anatomical parameters of the *Calamagrostis epigéjos* plants and the content of radionuclides show that the coefficients have a weak dependence or no communication. Power of internal irradiation doses are in the range of 40 to 760 µGy/day. These doses are insufficient for the occurrence of sustained changes in the anatomical structure of plants *Calamagróstis epigéjos*. This is confirmed by the literature data, which indicate that in the plant species most sensitive to radiation, the effects of chronic irradiation were observed at a dose rate of 1000 to 3000 µGy/h (24,000–72,000 µGy/day) [48].

Table 9 shows the correlation coefficients calculated to detect the possible influence of toxic elements on the anatomical parameters of plants.

The results of the calculation of correlation coefficients show that there is a connection between parameters such as the anatomical parameters of the *Calamagróstis epigéjos* plants and the content of elements in samples of plants. A strong correlation is noted between the anatomical parameters of the plants, and chemical elements such as Al (0.8, –0.8), Cr (−0.8), Cd (−0.9), Pb (0.95, –0.7), Sr (0.8), U (0.7), Cu (−0.9), Be (0.8), Ni (0.8, −0.77), and Rb (–0.7). The lanthanides have a medium degree of correlation (from 0.5 to 0.7).

In the analysis of the tabular data, there were significant correlation coefficients set for the toxic elements, namely in 30% of examined cases. There is a correlation between the chemical elements and anatomical parameters such as leaf mesophyll and the epidermis of the stem.

The correlation and regression analysis of the concentrations of toxic elements and anatomical parameters of the leaves showed that between these options there is a link. This relationship is both directly proportional and inversely proportional. The relationship is described by a linear regression (Table 10).

**Figure 3:**
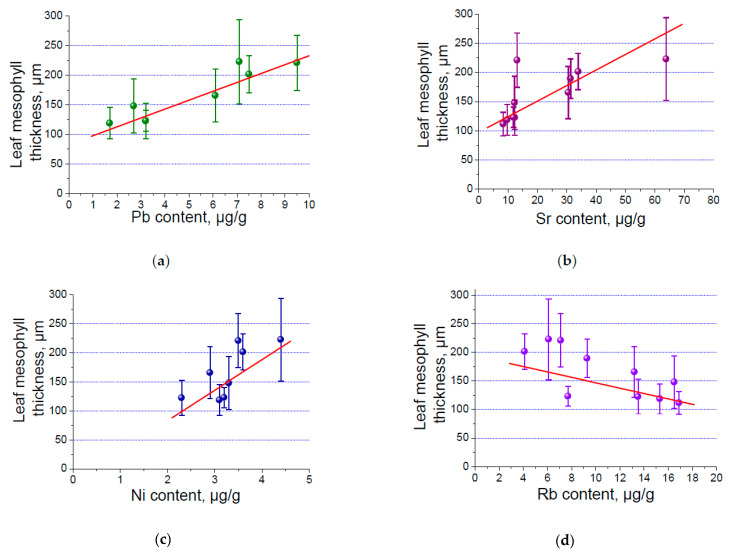
The the thickness of the mesophyll of a leaf from concentrations of toxic elements: Pb (**a**), Sr (**b**), Ni (**c**), and Rb (**d**).

The results show that there is a close relationship between the anatomical parameters of the leaves and elements such as aluminium, lead, strontium, uranium, nickel, samarium, and rubidium. A graphical example of the statistical analysis of the relationship of the described parameters is shown in Figure 3.

Figure 3 shows that the thickness of the mesophyll of the leaf blades increases with an increase in the concentration of elements such as Pb, Sr, and Ni. The value of the anatomical parameter of Pb has a high power direct correlation (R^2^ = 0.7). In other cases, the proportional relationship is of medium strength. Thus, we can assume that the mesophyll of the leaves increases with an increase in the concentration of elements such as Pb, Sr, and Ni.

The thickness of the mesophyll of a leaf decreases with increase in the concentration of Rb. The value of this parameter has a moderately strong inverse relationship (R^2^ = 0.6) with the content of Rb (Table 11).

The results show that there is a close relationship between the anatomical parameters of the stems and elements such as aluminium, cadmium, uranium, copper, beryllium, nickel, samarium, and iron. The most significant results will be presented as a graphic example.

**Figure 4:**
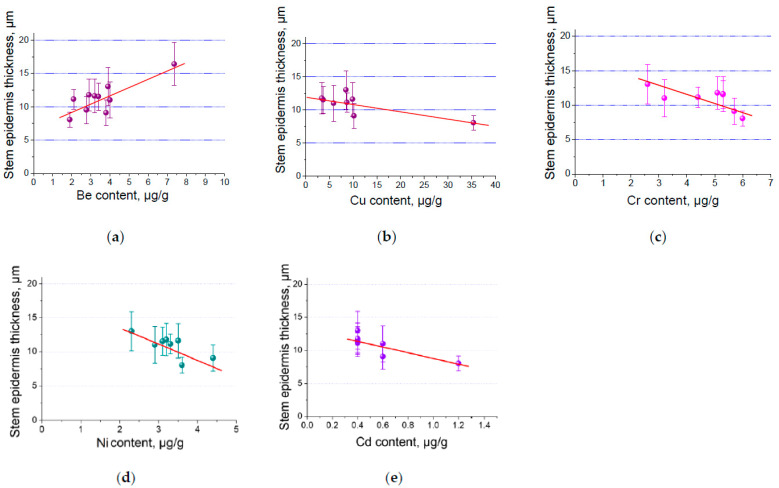
The thickness of the epidermis of the stem on the concentration of the elements Be (**a**), Cu (**b**), Cr (**c**), Ni (**d**), and Cd (**e**).

Figure 4 shows a direct dependence of the values of the epidermis of the stems on the content of Be in plants. The value of this parameter has a high power dependence (R^2^ = 0.7) from the element Be. The inverse dependence of the values of the epidermis of the stems is determined from the concentration of elements such as Cd, Cu, Cr, and Ni. The value of this anatomical parameter has a medium and highly inverse relationship (R^2^ = 0.6 and 0.7) with the content of the elements listed. Thus, we can assume that the increase in toxic elements adversely affects the anatomical structure of plants. In this case, the epidermis of the stem is reduced. These results confirm the data of authors who have published earlier works on the morphological and anatomical structure of plants [21,25,49,50]. According to their work in conditions of soil pollution with elements, it was found that anatomical structures such as the epidermis and the size of the parenchyma are smaller compared to plants growing in clean areas.

The results of these studies showed that there is an influence of toxic elements on the anatomical structure of leaves and stems at the histological level. The relation of the plant response in terms of anatomical parameters depends on the pollutant.

## 3. Materials and Methods

### 3.1. Experimental Site

The research was conducted at the former “Degelen” test site. The “Degelen” testing site is located in the similarly named low-mountain range Degelen, and is one of the main testing areas of the STS. Underground nuclear explosions were performed in tunnels, which are a horizontal mine with an average cross-section of about 9 m^2^, and a distance in depth from 500 to 1000 m. Each tunnel ended with an end box, in which an explosive charge was placed. A total of 181 tunnels were built at the “Degelen” site, where 209 underground nuclear explosions were conducted during the period from 1961 to 1989. The maximum explosive yield was 20 kT [36,37].

As a result of underground nuclear blasts, rocks became more permeable owing to the deformation of rock mass producing extensive cavities, zones of man-made fracturing, and space in tunnels themselves. Radionuclide-contaminated waters, while moving through crack systems and the tunnel cavity, recharge the basin of ground waters or outflow to the surface in the vicinity of a tunnel entry [36,37]. Tunnel No. 504, characterized by the presence of a watercourse in the form of a stream, was chosen for the study of the morpho-anatomical parameters of the plants.

At the experimental site of tunnel No. 504, the sites were selected with minimum and maximum values of radiative parameters (flux density β-particles and the equivalent dose rate (EDR)). Measurements of radiation parameters were carried out at a height of 0–5 cm from the soil surface. The measurements were performed using a dosimeter–radiometer MKS-AT6130 (production of Belarus). Values of equivalent dose on the study area vary in the range of 0.7 to 32 µSv/h, and the flux density of β-particles ranged from 10 to 1000 particles/min × cm^2^. Thus, the 10 study sites were chosen in areas with different levels of equivalent dose rate and flux density of beta-particles. Each study site consisted of a plot with an area of 1 m^2^. The distance between sites was 50 m. The dominant *Calamagrostis epigejos* was selected as the experimental plant, which grew on all research sites.

#### 3.1.1. Sampling for Morphological Studies

Above-ground parts of plants in each research site were selected for the morphological studies. Shoots of the current year of life were selected by mowing during the vegetation phase, i.e., flowering, which is approximately 90–100 days after germination. All shoots were sampled directly from the watercourses. The study selected the morphological parameters of plant height, stem length, length of panicle, and length of the leaf.

#### 3.1.2. Sampling for Anatomical Study

For the anatomical studies, fragments of bodies were selected from each plant: the stem was 2 cm with the first internode at 3 cm (the part of the stem between the first and second internodes), 4 cm from the second internode, the median part of the leaf. Samples of each plant were placed in glass containers with a volume of 20 mL, which were filled with the fixing balm. The balm was prepared immediately before the fixation of the material by mixing equal proportions of alcohol, glycerine, and distilled water. The samples were tightly covered with a lid. Anatomical preparations were made in accordance with conventional methods [51].

#### 3.1.3. Sampling of Plants for Radionuclide and Elemental Analyses

Above-ground parts of plants in each of the research sites were stripped by the method of hay harvest for elemental and radionuclide analyses. Samples were placed separately from each other in plastic bags and provided with a label listing the radiometric parameters of each site [52,53]. Packaged samples taken to the laboratory.

### 3.2. Analytical Work

#### 3.2.1. Preparing Plant Samples for Anatomical Research

The micro-specimens were obtained from fixed samples for their direct examination under a microscope. The slices were obtained with a microtome “TECHNOM” (Ekaterinburg, Russia). A fragment of stem or leaf was placed on a freezing microtome device. The thickness of the cut plant samples were dependent on the state of the plant and the coarseness of the fibers. The thickness of the sections of the stems was in the range of 20–30 mm, and leaves of 30–60 µm. Slices were carefully washed off with a knife on a glass slide with distilled water and replaced it on the glycerin. The cover glass was put on top [54].

**Figure 5:**
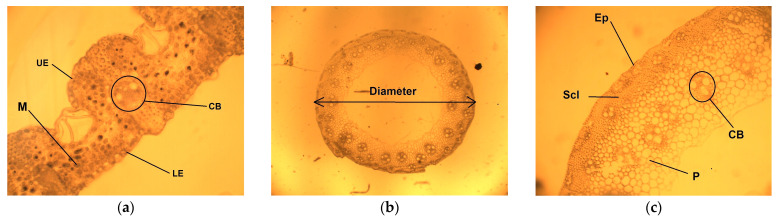
A cross-section of the leaf, an increase of 20×, UE—upper epidermis, LE—lower epidermis, M—mesophyll, CB—conductive bundle (**a**). A cross-section of the stem, an increase of 4×: diameter of the stem (**b**); a cross-section of the stem, an increase of 10×: Ep—epidermis, Scl—sclerenchyma, CB—conductive bundle, P—parenchyma (**c**).

The resulting slices were examined with a Micros MC 300 microscope with a camera. Each slice was investigated with increasing magnification of 4, 10, 20, 40 and 100×. A camera Vision Cam V500/21 M took the pictures necessary for the micro-preparations. Measurements of sample parameters were conducted using the BioWizad 4.2 software. The studied parameters of the leaves of *Calamagróstis epigéjos* were the thickness of the upper and lower epidermis, the thickness of mesophyll, and the area of the conductive bundles (Figure 5a); the measured parameters of the stem were the diameter of the stem (Figure 5b), the thickness of the epidermis, the thickness of the sclerenchyma, and the area of the conductive bundles located in the parenchyma (Figure 5c).

#### 3.2.2. Sample Preparation and Radionuclide Analysis

The process of preparing plants for radionuclide analysis included the grinding, homogenization, and weighing of the sample. Determination of the activity of ^137^Cs, ^241^Am, ^90^Sr was carried out instrumentally, so the samples in a dry powdered form were sent for analysis.

The analysis of the activity of ^239+240^Pu in the samples of plants was carried out with preliminary radiochemical preparation. Preparation included the ashing of the sample weight of 2 g at a temperature of t = 550 ± 5 °C for 7–8 h until ashing was complete. Furthermore, ashes of the sample were supplied to the acid digestion with subsequent extraction—chromatographic separation of isotopes of plutonium and by the electrolytic deposition of radionuclides on the stainless disk. Loss control was carried out by depositing a sample of a radioactive tracer ^242^Pu.

The analysis of the specific activity of radionuclides ^137^Cs and ^241^Am was carried out by gamma-spectrometer using a high purity germanium detector BE3830 manufactured by Canberra. The analysis of the activity of ^90^Sr was performed using the scintillation beta spectrometer Progress. Analysis of the counting of samples of ^239+240^Pu was conducted on an Alpha Analyst alpha spectrometer produced by Canberra. The results were from the measurements of the activity in samples of plants raised at the initial weight using the coefficients of drying and ashing.

Typical detection limits for wet-weight samples of ^137^Cs plants were 1 Bq/kg, ^241^Am—3 Bq/kg, ^239+240^Pu—0.3 Bq/kg, and ^90^Sr—100 Bq/kg.

#### 3.2.3. Sample Preparation and Elemental Analysis

Sample preparation included weighing (wet-weight), rinsing the sample with distilled water, drying, grinding, homogenization, dry weight determination, and sifting through a sieve with a diameter of 0.2 mm. Then, suspension samples weighing 0.5 g were selected and digested by the method of acid digestion.

The acid digestion of plant samples for elemental analyses included the following: suspension of the sample mass of 0.5 g of dried (chopped) analytical sample was placed in the reaction beaker, wetted with distilled water, then nitric acid was added in an amount of 5 mL. Then the weighed sample was transferred into an autoclave and heated in the laboratory oven for 30 min. The heated autoclave was placed in a drying cabinet heated to a temperature of 160 ± 2 °C for 2.5 h. At the end of the digestion process, the containers were cooled to room temperature, after which the autoclave was added to 1.5 cm^3^ of hydrogen peroxide.

The resulting solution was again dried in a drying cabinet or 1.5 h and, after cooling to room temperature, it transferred to a volumetric flask, brought to a volume of 15 cm^3^ with a solution of 1% nitric acid, and packed in polypropylene tubes with screw caps (vials). In case of sediment, the solution was centrifuged, and the supernatant was transferred into a measuring test tube and brought to a volume of 15 cm^3^ 1% with a solution of nitric acid, mixed and transferred into vials.

Heavy metal and toxic element contents were determined using a quadrupole by the inductively coupled plasma mass-spectrometer Elan 9000 by the “Perkin Elmer SCIEX” company complete with a PC and specialized software. The spectrometer calibration used 10- and 20-µg/L calibrating solutions. Multielement reference standard solutions (RS) containing metals made by Perkin Elmer (Waltham, MA, USA) were used for calibration with a rated certified value of metal content equal to 10 mg/L with the uncertainty of a certified value of 0.5% (dilution factor k = 2). Measurement quality was verified by measuring the calibrating solution of every 10 samples. To prepare accuracy control samples (validation) for calibration characteristics, RS Inorganic Ventures IV-ICP-MS-71A, CMS-1 (Inorganic Ventures, Christiansburg, VA, USA) containing metals were used with a rated certified value of metal content equal to 10 mg/L^−1^ and the uncertainty of a certified value equal to 0.5% (dilution factor, k = 2). If a calibration result was unsatisfactory (deviation of the calibration graph by 8–10%), the instrument was recalibrated taking into account new background parameters.

The analysis was carried out to determine the content of elements according to the ISO 17294-2׃2003 (E) procedure (state registration number is 022/10505 dated 27.12.05). Plant samples were analyzed, and the content of the following elements was determined: Al, Li, Be, Cr, Mn, Fe, Co, Ni, Cu, Zn, Sr, Y, Mo, Cd, Cs, Ba, La, Ce, Pr, Nd, Sm, Gd, Tb, Dy, Ho, Er, Tm, Yb, Lu, Re, Bi, U, V, Pb, and Sc. The analysis was carried out according to the ISO 17294-2׃2003 (E) procedure (state registration No. 022/10505 dated 27.12.05).

### 3.3. Evaluation of Radiation Doses

The methodology for the evaluation of doses to biota is described in detail in ICRP publication No. 108 [55]. There are several types of organism living in the water, in the ground and above ground. In our case, the interest is in wild grass. Because of the variability of living organisms in the natural environment, it is impossible to cover all the conditions of radiation exposure. In this regard, dosimetric models are based on the typical dimensions of the body.

The absorbed dose of internal and external exposure of 1 Bq of activity of a radionuclide in the soil or in the plant (dose coefficients) is calculated according to the expressions:

(1) $$\begin{array}{l} DCC_{Int}=\sum_{\nu }\left(\sum_{i}E_{i}\cdot Y_{i}\cdot \phi_{v}(E_{i})+\int N_{\nu }(E)\cdot E\cdot \phi_{\nu }(E)\cdot dE\right)\\ DCC_{Ext}=\sum_{\nu }\left(\sum_{i}E_{i}\cdot Y_{i}\cdot \left(1-\phi_{v}(E_{i})\right)+\int N_{\nu }(E)\cdot E\cdot \left(1-\phi_{\nu }(E)\right)\cdot dE\right) \end{array}$$

where *ν* denotes radiation type (alpha, beta, and gamma radiations, and spontaneous fission fragments); *E_i_* (MeV) and *Y_i_* (per decay) are energy and yield, respectively, of the discrete energy radiations per decay of the radionuclide; *N_ν_* (*E*) (per decay per MeV) is the energy spectrum for continuous energy radiations of type *ν* (here, for beta particles alone); and *Φ_V_* (*E*) is the absorbed fraction. A key quantity for estimating internal dose is the absorbed fraction, *Φ_V_* (*E*), which is defined as the fraction of energy emitted by a radiation source that is absorbed within the target tissue, organ, or organism. If organism size is small compared to gamma-quantum or particle path, then the internal dose decreases as a consequence. And, on the contrary, for beta particles of low energy and alpha particles, the range in the target tissue is extremely short, therefore it tends to a unity for the absorbed energy. Dimensions of dose factors are µGy × kg/day/Bq.

A similar approach is described in the publication of the United Nations Scientific Committee on the effects of atomic radiation—“Methodology of dose assessment for the biota” [56]. Dose coefficients of ICRP publication No. 108 are recommended for the calculation of radiation doses to the biota. The assessment of radiation doses to biota is performed through dose coefficients.

In this case, the internal and external dose rates are estimated as [57]:

(2) D=A×DCC

where *A* is the specific activity of a crude sample of the plant or the specific activity of the underlying soil (Bq/kg), *DCC*—the dose coefficient of internal or external exposure—Table 12 (µGy × kg/day/Bq).

For the radionuclides in question, ICRP regulates the following dose factors for internal and external exposure (Table 12).

## 4. Conclusions

- The study of the morphological parameters of the stems and leaves showed that stem length varied from 79 ± 10 to 120 ± 9 cm, leaf length from 25 ± 8 to 49 ± 4 cm, length of panicles from 23 ± 6 to 32 ± 5 cm, and plant height from 102 ± 6 150 ± 9. These values correspond to the normal morphological values.
- Studies have found that elevated levels of ^137^Cs (from 0.1 × 10^4^ ± 0.02 × 10^4^ to 4.9 × 10^4^ ± 0.5 × 10^4^ Bq/kg) and ^90^Sr (0.2 × 10^4^ ± 0.1 × 10^4^ to 6.2 × 10^4^ ± 0.5 × 10^4^ Bq/kg) in plants and the total absorbed dose (40–760 µGy/day) from all radionuclides did not have a significant effect on the anatomical parameters of plants *Calamagróstis epigéjos*.
- This experiment established that the toxic elements affect the plants more than the radionuclide. The results of the elemental analysis obtained data on the relations between the anatomical characteristics of studied plants and the content of such elements as Al, Pb, Sr, Cr, Cd, U, Cu, Be, Ni, Rb, Sm, and Fe.
- Variations in anatomical parameters of leaves and stems occurred at elevated concentrations of elements: for leaves—Al, Pb, Sr, U, Ni, Rb, and Sm, and for stems—Al, Cr, Cd, U, Cu, Be, Ni, Sm, and Fe.

A conductive bundle of the leaves decreased from 17,000 ± 1000 µm^2^ to 10,000 ± 2000 µm^2^, the mesophyll of the leaves decreased from 220 ± 47 to 110 ± 20 µm in the case the concentration of such elements as Al (130–490 µg/g), and Rb (4–17 µg/g) increased. The increase in the content of Pb (1.7–9.5 µg/g), Sr (8–64 µg/g), U (1–9 µg/g), Ni (2–4 µg/g), and Sm (0.1–0.5 mg/g) affected the increase in the mesophyll of the leaves (from 110 ± 20 to 220 ± 47 µm), the lower epidermis (from 16 ± 2 to 25 ± 5 µm), and the upper epidermis (from 9 ± 2 to 18 ± 5 µm).

The area of the conductive bundle of the stem increased from 11,000 ± 2000 to 18,000 ± 3000 µm^2^ as Al concentration increased from 130 to 490 µg/g, and Fe increased from 95 to 600 µg/g. The increase in the concentration of Cr (3–6 µg/g), Cd (0.4–1.2 µg/g), Cu (3–35 µg/g), and Ni (2–4 µg/g) had an impact on the decrease in the epidermis of the stem (16 ± 3.2 to 8.0 ± 1.1 µm). The diameter of the stem increased (from 10,000 ± 3000 µm to 19,000 ± 3000 µm) at an elevated concentration of U (1–9 µg/g) and Sm (0.1–0.5 µg/g).

- Such anatomical parameters of plants as the mesophyll of the leaf and epidermis of the stem are the most sensitive to the effects of toxic elements. Other anatomical parameters are exposed to toxic elements to a lesser degree.
- Parameters of plants such as the leaf mesophyll and the epidermis of the stem are recommended for use in studies of the indicative parameters of plants growing under conditions of chemical environmental pollution.
- Thus, the obtained data can be used to assess the environmental situation in the study area. The results can also serve as the input parameters of models used for the risk assessment of the impact of chemical pollution on biota.

## Figures and Tables

**Table 1:** Measuring morphological parameters of the leaves and stems of *Calamagróstis epigéjos*.

Site Number	Morphological Parameters (*n =* 20–25)			
	Stem Length, cm	Leaf Length, cm	Panicle Length, cm	Height, cm
1	79 ± 10	45 ± 5	24 ± 7	102 ± 6
2	87 ± 8	45 ± 6	28 ± 3	115 ± 7
3	120 ± 14	25 ± 8	28 ± 3	150 ± 13
4	118 ± 13	41 ± 12	25 ± 6	147 ± 13
5	106 ± 13	44 ± 5	23 ± 6	129 ± 10
6	95 ± 4	45 ± 6	21 ± 3	115 ± 5
7	89 ± 7	49 ± 4	24 ± 3	113 ± 9
8	89 ± 10	49 ± 12	23 ± 5	111 ± 13
9	120 ± 9	47 ± 4	32 ± 5	150 ± 9
10	114 ± 7	46 ± 5	27 ± 3	142 ± 9
Literature data [41]	50–120	Up to 50	Up to 30	80–150

Thus, in 10 study sites, stem length varied from 79 ± 10 to 120 ± 9 cm, sheet length from 25 ± 8 to 49 ± 4 cm, length of panicles from 23 ± 6 to 32 ± 5 cm, and plant height from 102 ± 6 to 150 ± 9 cm.

**Table 2:** The results of the measurements of anatomical parameters of the leaves and stems of *Calamagróstis epigéjos*.

Point No.	Anatomical Parameters (*n* = 20–25)							
	Leaf				Stem			
	The Thickness of the Epidermis on the Lower Side, µm	The Thickness of the Epidermis from the Upper Side, µm	Mesophyll Thickness, µm	The Area of the Conductive Bundle (103), µm^2^	The Diameter of The Stem (103), µm	The Thickness of the Epidermis, µm	The Thickness of the Sclerenchyma, µm	The Area of the Conductive Bundle in Parenchyma (103), µm^2^
1	25 ± 5	18 ± 5	220 ± 70	12 ± 4	3.3 ± 0.2	9 ± 2	100 ± 11	13 ± 3
2	17 ± 2	11 ± 2	200 ± 30	14 ± 4	2.0 ± 0.2	8 ± 1	110 ± 10	14 ± 3
3	22 ± 4	13 ± 2	110 ± 20	10 ± 2	2.3 ± 0.1	10 ± 2	80 ± 14	12 ± 2
4	18 ± 3	9 ± 2	170 ± 45	10 ± 2	2.2 ± 0.1	11 ± 3	110 ± 13	18 ± 3
5	16 ± 2	13 ± 2	190 ± 30	19 ± 3	2.1 ± 0.1	16 ± 3	110 ± 12	11 ± 3
6	17 ± 2	11 ± 2	120 ± 30	10 ± 3	2.8 ± 0.2	13 ± 3	120 ± 15	16 ± 3
7	22 ± 3	12 ± 2	150 ± 30	17 ± 1	2.3 ± 0.1	11 ± 2	80 ± 9	11 ± 2
8	19 ± 3	12 ± 2	120 ± 20	13 ± 2	2.2 ± 0.1	12 ± 2	100 ± 10	12 ± 2
9	18 ± 3	16 ± 5	220 ± 47	13 ± 4	2.6 ± 0.1	12 ± 3	110 ± 13	14 ± 3
10	20 ± 5	14 ± 1	120 ± 15	12 ± 4	3.0 ± 0.1	12 ± 2	100 ± 8	14 ± 2

**Table 3:** The results of the statistical analysis of the anatomical parameters of the leaves of *Calamagróstis epigéjos*.

Site No.	1	2	3	4	5	6	7	8	9	10
Anatomical parameter	The epidermis on the lower side of the leaf									
The range of variation, µm	18–35	12–21	14–27	13–25	11–24	13–21	17–27	14–25	14–26	11–28
Average, µm	25	17	22	18	16	17	22	19	18	20
Median, µm	24	17	23	17	16	17	22	18	18	19
Standard deviation, µm	5	2	4	3	3	2	3	3	3	5
The coefficient of variation, %	19	13	16	19	21	11	14	16	17	23
The index of asymmetry	0.7	−0.1	−0.8	0.6	0.5	−0.4	−0.05	0.6	0.8	0.1
Anatomical parameter	The epidermis on the upper side of the leaf									
The range of variation, µm	12–31	8–15	11–19	5–12	90–17	8–15	9–15	10–17	10–27	11–16
Average, µm	18	11	13	9	13	11	12	12	16	14
Median, µm	16	11	13	10	13	12	12	12	15	14
Standard deviation, µm	5	2	2	2	2	2	2	2	5	1
The coefficient of variation, %	30	18	15	20	17	17	16	16	33	9
The index of asymmetry	1.0	0.2	1.3	−0.6	0.1	0.3	0.2	1.0	0.6	0.3
Anatomical parameter	Mesophyll									
The range of variation, µm	150–340	160–240	70–150	100–250	140–250	80–180	120–210	80–160	130–320	100–160
Average, µm	220	200	110	170	190	120	160	120	220	120
Median, µm	200	210	110	150	180	110	140	110	210	120
Standard deviation, µm	71	31	20	45	34	30	30	24	47	14
The coefficient of variation, %	32	15	18	27	18	25	19	21	21	11
The index of asymmetry	0.5	−0.3	−0.2	0.5	0.3	0.8	0.8	0.2	0.3	1.6
Anatomical parameter	Conducting bundle									
The range of variation, µm	7100–21,000	7600–18,500	8400–14,000	7600–13,000	15,000–24,000	7000–13,000	15,000–20,000	10,000–17,000	8500–19,000	5500–15,000
Average, µm	12,000	13,500	10,000	9700	19,000	10,000	17,000	13,000	13,000	12,000
Median, µm	11,000	14,800	9000	9500	18,500	11,000	17,000	13,000	13,000	12,000
Standard deviation, µm	3800	3450	2400	2100	2900	2500	1400	2300	3800	3600
The coefficient of variation, %	32	25	23	22	15	25	8	17	29	31
The index of asymmetry	1.3	−0.6	1.3	0.7	0.3	−0.6	−0.1	0.4	0.3	−0.6

**Table 4:** The results of the statistical analysis of the anatomical parameters for stems of *Calamagróstis epigéjos*.

Site No.	1	2	3	4	5	6	7	8	9	10
	The diameter of the stem									
The range of variation, µm	3100–3700	1800–2700	2100–2400	2100–2300	2000–2300	2400–3100	2000–2500	2100–2300	2400–2800	2900–3100
Average, µm	3300	2000	2300	2200	2100	2800	2300	2200	2600	3000
Median, µm	3300	1900	2300	2200	2200	2800	2300	2200	2600	3000
Standard deviation, µm	150	240	130	70	100	250	130	50	140	50
The coefficient of variation, %	5	12	6	3	5	9	6	2	5	2
The index of asymmetry	0.6	1.3	0.01	−0.2	−0.3	−0.2	−0.7	−0.2	0.1	−0.2
	The epidermis									
The range of variation, µm	6–14	7–10	6–15	6–16	10–22	9–20	9–15	7–15	8–17	9–17
Average, µm	9	8	10	11	16	13	11	12	12	12
Median, µm	8	8	9	11	17	12	11	12	11	12
Standard deviation, µm	2	1	2	3	3	3	2	2	3	2
The coefficient of variation, %	21	14	21	25	20	22	13	18	22	20
The index of asymmetry	1.0	0.6	0.9	0.01	−0.3	0.7	1.1	−0.7	0.4	0.7
	Sclerenchyma									
The range of variation, µm	85–120	90–130	50–100	80–130	90–130	85–140	70–100	80–120	85–130	80–120
Average, µm	100	110	84	110	110	120	80	100	110	100
Median, µm	110	110	86	110	110	120	80	100	110	100
Standard deviation, µm	11	9	14	13	12	15	9	10	13	8
The coefficient of variation, %	11	8	17	12	11	12	11	10	12	8
The index of asymmetry	−0.2	−0.2	−1.2	0.2	0.2	−0.3	0.9	0.4	−0.7	−0.5
	The conductive bundle									
The range of variation, µm	7300–19,400	8500–21,000	7800–16,000	13000–25,400	5000–16,000	9000–21,000	9000–15,000	9000–17,000	8900–20,000	9500–18,000
Average, µm	13,000	14,000	12,000	18,000	11,000	16,000	11,000	12,000	14,000	14,000
Median, µm	13,000	14,000	12,000	18000	11,000	17,000	11,000	12,000	14,000	14,000
Standard deviation, µm	3200	3200	2400	34,00	2700	3100	1500	2200	2600	2200
The coefficient of variation, %	24	23	20	19	26	19	14	18	19	15
The index of asymmetry	0.03	0.2	−0.1	0.7	−0.2	−0.6	0.6	0.4	0.3	−0.5

**Table 5:** The results of the determination of the specific activity of radionuclides in plants.

Point No.	The Specific Activity of Radionuclides, Bq/kg						
	^40^K	^226^Ra	^232^Th	^241^Am	^239+240^Pu	^137^Cs (*n* × 10^4^)	^90^Sr (*n* × 10^4^)
1	170 ± 30	19 ± 4	47 ± 9	<1.5	1.4 ± 0.8	1.5 ± 0.2	2.1 ± 0.3
2	260 ± 50	<4	8 ± 4	<3	1.3 ± 1.0	0.1 ± 0.02	0.6 ± 0.1
3	<75	<8	33 ± 7	<11	<0.3	4.5 ± 0.4	0.5 ± 0.1
4	310 ± 60	<7	24 ± 5	<4	<1.1	4.9 ± 0.5	1.6 ± 0.2
5	<50	<6	<6	<3	<0.14	1.5 ± 0.2	1.2 ± 0.1
6	160 ± 30	<7	<6	<4	<0.3	1.4 ± 0.2	0.5 ± 0.1
7	230 ± 50	<9	<7	<5	<0.3	1.9 ± 0.3	0.5 ± 0.1
8	300 ± 60	<4	<3	<4	2.3 ± 0.1	2.6 ± 0.3	3.4 ± 0.3
9	190 ± 40	<4	11 ± 3	<3	<1.03	0.4 ± 0.1	6.2 ± 0.5
10	170 ± 30	4 ± 2	<3	<1.1	0.6 ± 0.3	0.3 ± 0.1	0.2 ± 0.1

**Table 6:** The results of the calculation of the dose of internal exposure of plants.

Point No.	Dose Rate, µGy/day	Point No.	Dose Rate, µGy/Day
1	310	6	110
2	70	7	120
3	210	8	490
4	360	9	760
5	190	10	40

**Table 7:** The results of determination of elements in plants.

Element	Point No.										Normal Concentration Range [47], µg/g
	1	2	3	4	5	6	7	8	9	10	
	Element Concentration, µg/g										
Al	360	240	400	490	350	360	140	130	200	150	to 200.0
Cr	6	6	2	3	2	3	4	5	5	5	0.1–0.5
Zn	120	140	125	170	100	130	160	40	73	75	27–150
Cd	0.6	1.2	0.5	1	1	0.4	0.4	0.4	0.4	0.4	0.05–0.2
Pb	7	7	5	6	5	3	3	2	10	3	5–10
Sr	64	34	8	31.0	31	12	12	10	13	12	6–37
U	9	1	2	2	5	2	1	2	3	1	0.01–0.1
Cu	10	35	10	6	8	9	9	4	10	3	5–20
Mn	970	1540	1400	1450	1300	1430	1580	1500	2100	1730	20–300
Be	4	2	3	4	7	4	2	3	3	3	1–7
Li	7	21	2	3	2	2	62	2	10	4	to 3.0
Co	0.4	0.4	0.4	0.4	0.4	0.4	0.4	0.4	0.4	0.4	0.02–1.0
Ni	4	4	3	3	3	2	3	3	4	3	0.1–5.0
Rb	6	4	17	13	9	14	17	15	7	8	20–70
Y	3	1	1	1	-*	1	1	1	2	1	0.2–8
La	3	1	1	2	1	1	1	1	2	1	0.1–0.2
Ce	5	1	1	3	1	2	1	2	2	1	0.2–0.3
Nd	2	0.4	1	1	0.5	1	0.3	1	1	0.2	0.05–0.2
Sm	0.5	0.1	0.2	0.2	0.1	0.2	0.1	0.1	0.2	0.1	0.02–0.04
Fe	330	200	250	600	450	460	360	95	310	200	50–100
Gd	0.7	0.1	-*	0.3	-*	0.3	0.1	0.2	0.3	0.1	0.02–0.04

Note: * no data available.

**Table 8:** The results of the calculation of correlation coefficients between the anatomical parameters of *Calamagróstis epigéjos* and the content of radionuclides in plants.

Radionuclide	Lamina				Stem			
	Lower Epidermis	Upper Epidermis	Mesophyll	Conducting Bundle	Diameter of Stem	Epiderma	Sclerenchyma	Conducting Bundle
^137^Cs	0.2	−0.4	−0.4	−0.3	−0.3	−0.1	−0.4	0.2
^90^Sr	−0.1	0.5	0.5	0.001	0.1	0.03	0.2	−0.01
^241^Am	0.2	−0.2	−0.4	−0.2	−0.4	−0.2	−0.6	−0.2
^239+240^Pu	0.1	0.4	0.6	−0.3	0.2	−0.5	0.3	0.3
Dose rate, µGy/day	−0.1	0.4	0.4	−0.1	−0.03	0.02	0.1	0.03

**Table 9:** The correlation coefficients between the anatomical parameters of the plants and toxic elements.

Element	Leaf				Stem			
	Lower Epidermis	Upper Epidermis	Mesophyll	Conducting Bundle	Diameter of Stem	Epiderma	Sclerenchyma	Conducting Bundle
Al	−0.1	−0.2	0.3	−0.8	0.1	−0.1	0.5	0.8
Cr	0.3	0.5	0.5	0.5	0.1	−0.8	−0.2	−0.6
Cd	−0.2	−0.3	0.5	0.02	−0.4	−0.9	0.3	0.1
Pb	0.03	0.3	0.95	−0.2	0.1	−0.7	0.6	0.2
Sr	0.3	0.4	0.8	0.1	0.4	−0.2	0.3	0.1
U	0.6	0.5	0.6	−0.2	0.7	−0.3	0.1	−0.1
Cu	−0.3	−0.2	0.5	0.2	−0.4	−0.9	0.3	−0.03
Be	−0.4	0.1	0.2	0.4	−0.04	0.8	0.4	−0.2
Li	0.3	−0.1	0.1	0.4	−0.2	−0.2	−0.5	−0.3
Ni	0.5	0.7	0.8	0.3	0.5	−0.8	−0.2	−0.5
Rb	0.1	−0.4	−0.6	−0.1	−0.3	0.1	−0.7	−0.1
Y	0.5	0.6	0.6	−0.5	0.6	−0.3	0.4	0.2
La	0.5	0.6	0.6	−0.5	0.6	−0.2	0.4	0.2
Ce	0.5	0.6	0.6	−0.5	0.6	−0.2	0.3	0.2
Nd	0.5	0.6	0.6	−0.4	0.6	−0.3	0.3	0.2
Sm	0.7	0.7	0.6	−0.3	0.7	−0.4	0.2	0.1
Fe	−0.04	−0.6	0.1	−0.5	0.02	0.2	0.2	0.7
Gd	0.6	0.7	0.6	−0.4	0.6	−0.2	0.3	0.1

**Table 10:** The results of the statistical analysis of anatomical parameters of the leaves of *Calamagróstis epigéjos* and their elemental composition.

Element	Lower Epidermis		Upper Epidermis		Mesophyll		Conducting Bundle	
	R^2^	Regression Equation	R^2^	Regression Equation	R^2^	Regression Equation	R^2^	Regression Equation
Al	0.01	y = −0.0014x + 20	0.05	y = −0.005x + 14.2	0.07	y = 0.1x + 142	0.6	y = −13.0x + 16,000
Cr	0.12	y = 0.7x + 16	0.29	y = 1.3x + 7	0.25	y = 18x + 80	0.24	y = 870x + 8500
Zn	0.00	y = 0.003x + 19	0.21	y = −0.03x + 16	0.03	y = 0.2x + 145	0.003	y = −2.6x + 13,000
Cd	0.06	y = −2x + 21	0.07	y = −3x + 15	0.22	y = 75x + 120	0.001	y = 200x + 13,000
Pb	0.00002	y = −0.004x + 20	0.01	y = 0.3x + 12	0.7	y = 14x + 100	0.02	y = −150x + 14,000
Sr	0.08	y = 0.04x + 19	0.1	y = 0.05x + 12	0.5	y = 3x + 120	0.002	y = 9x + 13,000
U	0.6	y = 0.7x + 18	0.6	y = 0.9x + 11	0.32	y = 9x + 140	0.03	y = −150x + 13,000
Cu	0.08	y = −0.1x + 20	0.04	y = −0.06x + 14	0.24	y = 2x + 140	0.04	y = 41x + 12,000
Mn	0.22	y = −0.004x + 25	0.01	y = −0.001x + 15	0.003	y = −0.01x + 170	0.06	y = 1.7x + 10,000
Be	0.2	y = −1x + 22	0.01	y = 0.2x + 12	0.03	y = 5x + 140	0.2	y = 850x + 10,000
Li	0.06	y = 0.03x + 19	0.02	y = −0.02x + 13	0.006	y = 0.2x + 160	0.2	y = 71x + 12,300
Ni	0.5	y = 3x + 10	0.5	y = 3x + 2	0.6	y = 54x−13	0.10	y = 1100x + 9000
Rb	0.01	y = 0.06x + 19	0.1	y = −0.2x + 15	0.6	y = −240−7x	0.02	y = −85x + 14,000
Y	0.21	y = 2x + 18	0.37	y = 3x + 10	0.40	y = 43x + 110	0.21	y = −1500x + 15,000
La	0.21	y = 2x + 18	0.32	y = 2x + 10	0.31	y = 35x + 120	0.23	y = −1500x + 14,500
Ce	0.27	y = 0.7x + 18	0.36	y = 1x + 11	0.30	y = 18x + 130	0.21	y = −130x + 14,000
Nd	0.30	y = 2x + 18	0.36	y = 3x + 11	0.35	y = 45x + 130	0.18	y = −1600x + 14,000
Sm	0.5	y = 13x + 17	0.43	y = 14x + 11	0.35	y = 190x + 130	0.11	y = −5200x + 14,000
Fe	0.002	y = −0.0006x + 18	0.10	y = −0.01x + 15	0.01	y = 0.03x + 160	0.24	y = −6.6x + 15,000
Gd	0.34	y = 8x + 18	0.44	y = 11x + 10	0.32	y = 140x + 130	0.16	y = −4700x + 14,000

Note: R^2^—a determination coefficient.

**Table 11:** The results of the statistical analysis of the anatomical parameters of the stems and their elemental composition.

Element	Stem Diameter		Epiderma		Sclerenchyma		Conducting Bundle	
	R^2^	Regression Equation	R^2^	Regression Equation	R^2^	Regression Equation	R^2^	Regression Equation
Al	0.01	y = 0.4x + 2400	0.01	y = 11 – 0.001x	0.23	y = 0.04x + 94	0.7	y = 13x + 11,000
Cr	0.01	y = 28x + 2400	0.5	y = 15 – 0.9x	0.03	y = 110 – 1.5x	0.40	y = 19,000 – 1100x
Zn	0.03	y = 2700 – 2x	0.08	y = 12 – 0.01x	0.01	y = 110 – 0.01x	0.12	y = 16x + 12,000
Cd	0.13	y = 2900 – 580x	0.7	y = 14 – 5x	0.07	y = 10x + 98	0.01	y = 870x + 14,000
Pb	0.04	y = 30x + 2400	0.1	y = 13 – 0.3x	0.2	y = 2x + 90	0.1	y = 300x + 12,000
Sr	0.1	y = 9x + 2300	0.06	y = 12 – 0.03x	0.1	y = 0.2x + 98	0.004	y = 9x + 13,000
U	0.5	y = 120x + 2200	0.11	y = 11 – 0.2x	0.02	y = 0.5x + 102	0.02	y = 15,000 – 110x
Cu	0.14	y = 2700 – 16x	0.6	y = 12.0 – 0.1x	0.08	y = 0.3x + 100	0.001	y = 14,000 – 6x
Mn	0.12	y = 3300 – 0.5x	0.15	y = 0.002x + 8	0.00002	y = 0.0001x + 100	0.005	y = 15,000 – 0.5x
Be	0.002	y = 2500 – 12x	0.7	y = 1.2x + 7	0.2	y = 3x + 90	0.04	y = 15,000 – 300x
Li	0.04	y = 2500 – 4x	0.04	y = 12 – 0.02x	0.2	y = 110 – 0.3x	0.1	y = 14,000 – 40x
Ni	0.11	y = 240x + 1800	0.6	y = 17 – 2x	0.04	y = 110 – 3x	0.23	y = 20,000 – 1600x
Rb	0.1	y = 2800 – 30x	0.02	y = 0.1x + 11	0.4	y = 120 – 2x	0.01	y = 14,000 – 50x
Y	0.31	y = 380x + 2100	0.06	y = 12 – 0.6x	0.17	y = 7x + 96	0.04	y = 660x + 13,000
La	0.31	y = 350x + 2100	0.03	y = 11 – 0.4x	0.14	y = 5x + 97	0.05	y = 680x + 13,000
Ce	0.34	y = 200x + 2200	0.04	y = 11 – 0.2x	0.11	y = 3x + 100	0.03	y = 290x + 14,000
Nd	0.33	y = 440x + 2200	0.08	y = 12 – 0.7x	0.10	Y = 6x + 100	0.03	y = 600x + 14,000
Sm	0.5	y = 2200x + 2100	0.12	y = 12 – 4x	0.04	y = 20x + 100	0.00	y = 1100x + 14,000
Fe	0.0003	y = 0.05x + 2500	0.05	y = 0.002x + 10	0.03	y = 0.01x + 100	0.5	y = 9x + 11,000
Gd	0.40	y = 1500x + 2200	0.05	y = 11 – 2x	0.08	y = 16x + 100	0.01	y = 1200x + 14,000

Note: R^2^—a determination coefficient.

**Table 12:** Values of dose coefficients for plants, µGy × kg/day/Bq.

Radionuclide	Dose Factor, *DCC_Int_*	Dose Factor, *DCC_Ext_*
^90^Sr + ^90^Y	1.2 × 10^−2^	3.0 × 10^−9^
^137^Cs + ^137m^Ba	3.4 × 10^−3^	2.7 × 10^−3^
^239+240^Pu	7.1 × 10^−2^	3.1 × 10^−6^
^241^Am	7.7 × 10^−2^	7.9 × 10^−5^

## Data Availability

The original contributions presented in the study are included in the article; further inquiries can be directed to the corresponding author.
